# Manifestations of Body Image Distress in AYA Cancer Survivors: A Qualitative Analysis of Participants in an Expressive Writing Intervention

**DOI:** 10.1002/pon.70626

**Published:** 2026-10-12

**Authors:** Victoria Wytiaz, Amy Hecht‐Zizes

**Affiliations:** ^1^ Department of Internal Medicine Division of Oncology University of Michigan Ann Arbor Michigan USA; ^2^ School of Social Work University of Michigan Ann Arbor Michigan USA

**Keywords:** adolescent/young adult oncology, body image distress, expressive writing, trauma‐informed care

## Abstract

**Background:**

Body image distress is a major concern for cancer survivors and includes views on physical appearance and body functions. As body image is largely developed in adolescence and young adulthood with implications for self‐identity and social relationships, it may impact adolescent and young adult (AYA) cancer survivors in distinct ways.

**Aims:**

Explore the manifestations of body image distress in adolescent and young adult cancer survivors who participated in a body image‐focused expressive writing intervention.

**Methods:**

Semi‐structured interviews focusing on the body image experience were conducted and reviewed with a reflexive thematic analysis approach embedded within an interpretative phenomenological framework.

**Results:**

Qualitative analysis of 24 interviews revealed the following themes: Feasibility and Acceptability; Reconciling Intellectual and Emotional Understanding of Anticipated Body Image Changes; What We Gained and What We Lost; Body Image Distress as a Source of Guilt and Shame; We are a Community, but We Lack a Community.

**Conclusions:**

Discussion of body image distress represented an example of trauma‐informed care that allowed participants to build a small community within the study and regain a sense of self identity lost in the cancer experience. This approach informs suggestions for cancer survivorship care and adopting a trauma‐informed care approach during clinical or research environment. Future studies will endeavor to facilitate the adaptation of expressive writing to the needs of a given cancer survivor while building a community around those who seek to further their intellectual and emotional understanding of the ramifications of body image distress with peers.

## 1 Background

Body image distress is a major psychosocial concern for cancer patients, encompassing views on physical appearance as well as thoughts and feelings related to how one's body functions [1]. Significant alterations in appearance and body function can emerge with disease progression or with cancer‐directed treatment and supportive measures. One's body image is largely developed in adolescence and young adulthood with implications for self‐identity and social relationships [2, 3]. It is therefore unsurprising that body image concerns affect adolescent and young adult (AYA) cancer patients and survivors in distinct ways when compared to an older cancer patient population [4].

Despite this recognition, cancer‐related body image distress remains poorly managed due to lack of time in the clinical setting and lack of provider expertise [5, 6]. Body image distress in other populations, specifically patients with an eating disorder or body dysmorphic disorder, may be treated as part of those overarching diagnoses with intensive psychotherapy or pharmacological interventions. However, there are no recommended interventions for AYA cancer survivors who suffer from cancer‐related body image distress that does not meet criteria for an alternative diagnosis [7, 8]. To address this knowledge gap, we proposed an in‐home body image‐focused expressive writing program as an outlet for addressing body image distress in a way that eliminates constraints of clinical time and specialist availability.

Expressive writing was designed to promote the processing of a traumatic event by prompting participants to write briefly about thoughts and feelings related to that experience [9]. The primary purpose of this pilot study was to determine the feasibility and acceptability of an in‐home body image‐focused expressive writing intervention in AYA cancer survivors with body distress by both evaluating participant recruitment, retention and adherence rates while a secondary, exploratory aim was to characterize within‐ and between‐group changes over the 4‐week intervention period with regard to body image distress. This pilot study did demonstrate feasibility and a larger, phase II, randomized controlled trial is in development to explore the efficacy of the intervention.

In addition to recruitment, retention and adherence, semi‐structured interviews with pilot study participants provided evidence of feasibility and acceptability of the expressive writing intervention. The candor and enthusiasm with which the AYA cancer survivors shared their experience of body image distress throughout their journey was unexpected and profound. The richness and depth of these accounts from a unique and often under‐represented patient population was deemed worthy of further study. As such, we adopt a reflexive thematic analysis approach embedded in an interpretative phenomenological framework to explore the manifestations of body image distress in AYA cancer survivors.

## 2 Methods

### 2.1 Sample

The study employed an explanatory‐sequential mixed‐methods pilot study to assess feasibility and acceptability. Cancer survivors with clinically relevant body image distress were recruited from cancer clinics at the University of Michigan as well as through institutional social media campaigns. Participants were eligible if they were 15–39 years old, had a history of at least one prior cancer diagnosis, had completed all treatment at least three months prior to enrollment and reported a body image distress score of 10 or higher on the Body Image Scale (BIS) [10, 11]. Participants were ineligible if they planned to receive cancer treatment during the study period, had a life expectancy of 3 months or less, were actively receiving treatment for body image distress or anxiety with a recent change in regimen (within the past 8 weeks) or had a history of limb‐altering surgery or amputation.

The initial screening process included general study description as well as administration of the BIS to assess baseline body image distress. This was undertaken by authors A.H.Z. (female) and V.W. (female) and both authors shared their roles and connection to the study during this point of contact to promote transparency. A.H.Z. is an MSW with a research focus on social justice and community change while V.W. is an MD with a clinical focus in sarcoma and research interest in AYA oncology and these roles were shared with participants prior to engagement in the study. Prior to BIS administration, A.H.Z. and V.W. provided warning to participants that the content included could be sensitive or potentially triggering and for some participants, this resulted in additional follow‐up prior to screening to ensure emotional preparedness. This study was approved by the University of Michigan Institutional Review Board (HUM00239179) and all participants provided written informed consent.

### 2.2 Procedure

Participants were randomized 1:1 to either an expressive writing intervention arm or a control writing arm with weekly writing prompts designed to either probe participants' thoughts and feelings regarding body image and the cancer experience or designed to be emotionally neutral [12, 13, 14]. Writing prompts are shown in Table 1. Participants completed the BIS at baseline and at the conclusion of the 4‐week study period. Authors A.H.Z. and V.W. conducted weekly check‐in calls to assess adherence while also establishing rapport and trust. V.W. and A.H.Z. conducted semi‐structured interviews focusing on participant body image experience as well as perceived feasibility, acceptability and safety of the intervention. Interviews were conducted by Zoom over 45–60 minutes, recorded, and then transcribed by a HIPAA‐compliant transcription service. No other individuals were present during interview, and no repeat interviews took place. Interview guide is shown in Figure 1.

**TABLE 1:** Writing prompts.

Trial arm	Intervention group	Control group
Week 1	How did your body support you through cancer care? In what ways do you feel like it let you down?	Describe in detail the activities you completed in the 24 hours prior to this task.
Week 2	Can you imagine a time in the future when you feel comfortable in and with your body? How would that feel, and does it depend on your cancer status?	Describe in detail your current surroundings.
Week 3	Describe a time when you changed your behaviors based on thoughts about your body (avoided social events, etc.). What feelings did you have after this?	Review the plot of the last book you read/movie or television show you watched.
Week 4	How would you speak to a friend with negative thoughts about their body? Is this different than your internal dialogue either during cancer treatment or after?	Detail the events of your most recent vacation or trip away from home, from departure to arrival home.

**FIGURE 1:**
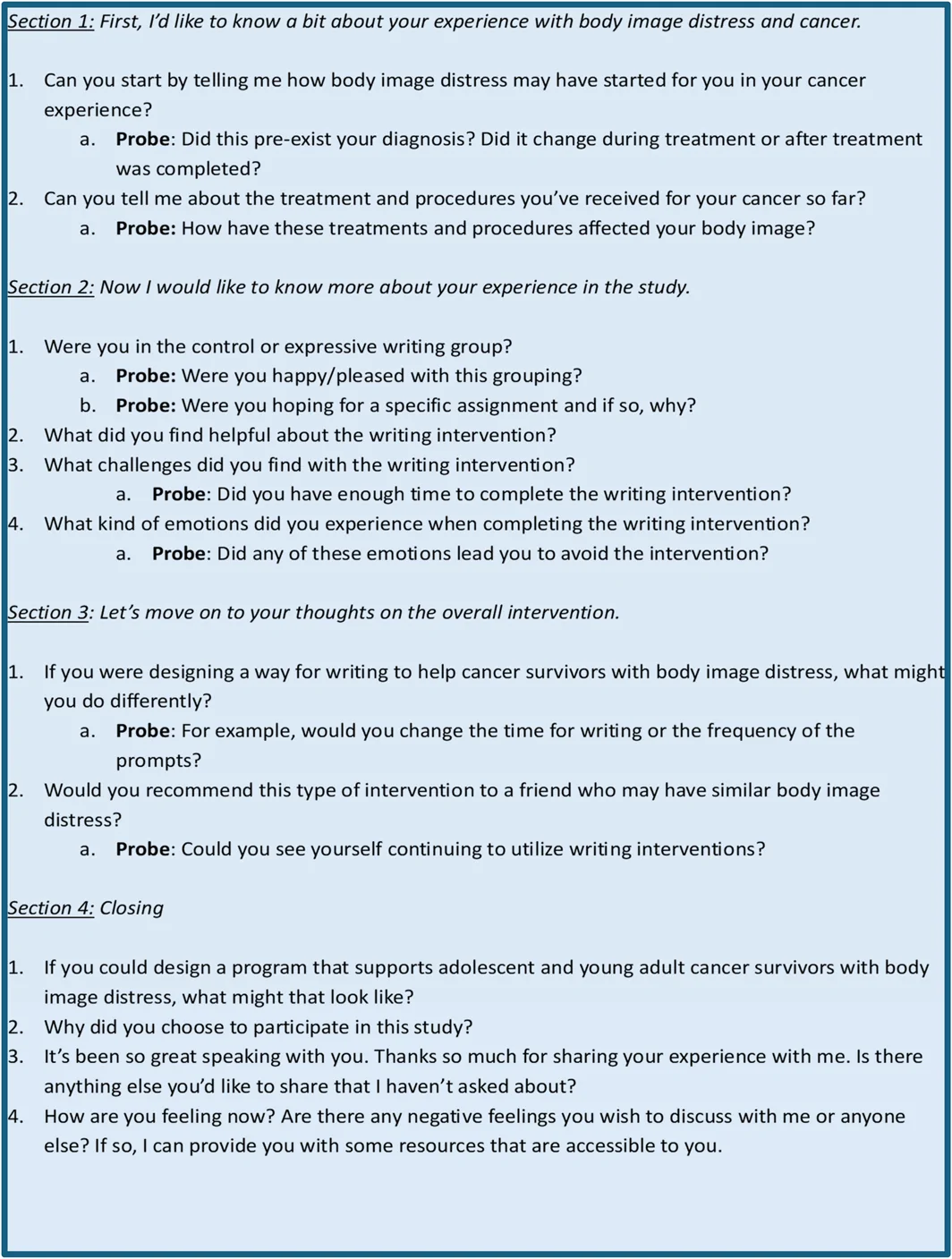
Interview guide.

### 2.3 Data Analysis

Following interviews, V.W. and A.H.Z. immediately documented initial impressions and following transcription, V.W. and A.H.Z. then analyzed the qualitative data using inductive content analysis with a specific focus on feasibility and acceptability of the expressive writing or control writing intervention [15]. V.W. and A.H.Z. independently read transcripts to derive codes, discussed labels to group codes into categories, and identified major themes and corresponding exemplary quotes. This content analysis was performed in isolation from the alternative analytic approach described below and was intended to explore the feasibility and acceptability of the proposed expressive writing intervention to further support larger‐scale randomized studies to evaluate the efficacy of the intervention.

In addition, authors V.W. and A.H.Z. undertook an alternative qualitative analytic approach to answer the question, “What is it like to experience body image distress as an AYA cancer survivor.” This is best defined as a reflexive thematic analysis approach embedded within an interpretative phenomenological framework. Participants from both the expressive writing intervention arm and control writing arm were included in this analysis as all participants were probed about their experience of body images distress as an AYA with cancer as well as their overall thoughts on supporting those with such distress throughout the cancer journey. As shown in Figure 1, there is a delineation of questions related to body image distress in general and those that are specific to the study.

#### 2.3.1 Interpretative Phenomenology and Reflexive Thematic Analysis in Concert

In considering the most appropriate context in which to explore the stated research question, A.H.Z. and V.W. were drawn to the epistemological stance of interpretative phenomenology in which the essences of a phenomenon are elucidated through participant accounts of that lived human experience [16, 17, 18]. In this endeavor, we consider the phenomenon of interest to be that lived experience of body image distress as an AYA cancer survivor, and the themes presented herein suggest the essences of this phenomenon. The initial interview structure adheres to interpretative phenomenology, which is inherently iterative, in that questions to participants were open‐ended and inspired long‐form narrative accounts and therefore rich data for analysis. Interpretative phenomenology is an ideal companion for our chosen analytic approach in that in interpretative phenomenology, there is the allowance for the coexistence of both the process of participants making sense of their lived experience of body image distress and the process of the authors A.H.Z. and V.W. to interpret that experience while recognizing their own assumptions and prior knowledge [19]. As noted above, A.H.Z. is an MSW with a focus on community change and as such, her analytical lens may place her interpretations of the presented data into a broader social justice landscape that transcends AYA oncology. Alternatively, V.W., as a medical oncologist in the sarcoma clinical space and AYA supportive oncology research field, brings the experience of witnessing manifestations of body image distress in her patients to her interpretation of the participant interview responses.

Rather than a hindrance, it is this recognition of unattainable objectivity that closely aligns interpretative phenomenology with reflexive thematic analysis. The term “reflexive” denotes actions that direct attention to the self (researcher) in a way that fosters a relationship between the research and the subject [20]. Reflexive thematic analysis is a flexible analytic approach that is useful for analyzing unstructured texts, such as long‐form interviews, to facilitate the discovery of previously identified factors associated with phenomenon of interest [21, 22, 23, 24]. Reflexive thematic analysis is a flexible analytic approach that shares with interpretative phenomenology the value of the research point of view and acknowledges how this shapes the presentation of the essences of the phenomenon. Furthermore, the practical steps outlined for proper reflexive thematic analysis are not dissimilar from those consistent with an interpretative phenomenology analysis [25, 26, 27]. For example, reflexive thematic analysis “data familiarization” mirrors the “reading and exploratory notes” phase of interpretative phenomenology. This agreement is also seen in “initial coding” and “formulating experiential statements” and “generation of initial themes” and “finding connections and clustering statements.”

Authors A.H.Z. and V.W. considered the experience of body image distress as an AYA cancer survivor to be a phenomenon of interest and endeavored to allow the participants in the study to present their own psychological processes and meaning making that body image distress while recognizing their own biases in interpreting and presenting that meaning. Following a holistic reading of interviews to derive the essence of the lived experiences described, the authors incorporated the steps of reflexive thematic analysis to organize themes for the final report [19]. To describe this process in brief, authors initially re‐familiarized themselves with the data through additional readings of the interview responses given the distinct goal of this process when compared to the initial content analysis and annotated these transcripts with initial thoughts relating to the body image distress experience of AYAs with a history of cancer. The generation of initial codes took place individually by A.H.Z. and V.W. and they met weekly after initial coding to review 2–4 interviews at each session. Authors also used these meetings to group these codes into potential themes, again working 2–4 interviews at a time. After the 24 interviews were coded and labeled with initial themes applied across the entire data set which led to naming and defining the themes in this final report with selected examples (i.e., quotations) providing support for this analysis.

#### 2.3.2 Rigor

We utilized multiple strategies to ensure the validity of this qualitative analysis. For this work, we define validity as accurately representing the lived experience of BID among the AYA cancer survivor participants [28]. As described in the above section detailing the interpretative phenomenological and reflexive thematic analysis approaches, researcher reflexivity allows for the identification of A.H.Z. and V.W. as social worker and medical oncologist, respectively, with their own background and experience in the care of AYAs with cancer. This disclosure allows the reader to interpret the analysis with an appreciation of what may be unintended but unavoidable biases. We also adopted a form of member‐checking in this study, specifically collaboration with participants as early interviews informed additional probing questions for subsequent interviews, often related to topics not previously considered relevant to BID in this population [29, 30]. For example, an early interview participant described a sense of pride in her surgical scars and as such, all further participants were asked if this resonated with them.

Rich descriptions are provided herein to provide vivid detail of the participant experience of BID and to contextualize that experience [31, 32]. Finally, peer debriefing provided the opportunity for author colleagues in the medical oncology, social work, adolescent and young adult mental health, and social justice spaces to both support and challenge the methods and interpretations of the accumulated data [33].

## 3 Results

Thirty participants were consented and 27 were randomized (expressive writing *n* = 14, control writing *n* = 13) in the overall study. 24 participants (23 F/1M, 23 white/1 black) engaged in virtual interviews as 3 were lost to follow‐up. 12 participants were randomized to the expressive writing arm while 12 were in the control writing arm. Among this subset of 24 participants, all 24 completed at least 3 of the 4 writing interventions as well as all baseline and end‐of‐study measures. These 24 participants also completed an Acceptability of Intervention Measure (AIM), a validated four‐item scale measured on a 5‐point Likert scale [34]. Mean AIM score in the expressive writing group was 3.4 compared to 2.9 in the control group.

Qualitative analysis of the content of the 24 interviews revealed the following themes: Feasibility and Acceptability; Reconciling Intellectual and Emotional Understanding of Anticipated Body Image Changes; What We Gained and What We Lost; Body Image Distress as a Source of Guilt and Shame; We are a Community, but We Lack a Community. Expressive writing or control writing arm assignment is indicated by EW or CW, respectively.

### 3.1 Themes

#### 3.1.1 Feasibility and Acceptability

Many participants felt that expressive writing fit into the busy lifestyles that many AYAs in the survivorship stage of cancer experience and appreciated the flexibility of the exercise.

In general, I love structure. But because of the craziness of life, I actually liked that [writing] was on my list of things to do. I liked having the freedom of you know, oh, there's something going on right now. I'm distracted. My brain's not in the best writing mode, so I'm not going to do it right this second.

[CW01]

AYAs found the private nature of the writing to be acceptable. One AYA shared that they “could really kind of flounder a bit and explore the prompts because it's not meant to be anything that anybody sees [EW01].”

Similarly, the act of writing for writing's sake and addressing questions of interest led to further expression of feasibility and acceptability. One participant described their writing process:

I like just writing because it is a way for me to get all of that overthinking and over‐explaining without someone saying, “you're overthinking” or dismissing it or saying, “why are you worried about that”? It's a way to validate it for me but to also be like, you need to get this out because it's not healthy to ruminate and be focusing on it.

[CW02]

#### 3.1.2 Reconciling Intellectual and Emotional Understanding of Anticipated Body Image Changes

Participants acknowledged that while there was an intellectual understanding that cancer and cancer treatments would alter the body, both physically and in terms of functionality, that did not necessarily correlate to emotional preparedness for such changes.

It is very overwhelming. No one really sat me down and went through the process step by step of this is what's going to happen. This is what it's going to look like. I mean, I saw pictures at my plastic surgeon's office of like, this is the “end product” when someone's completely healed, but I didn't see a picture of like, “this is what you're going to look like right after surgery”. Even I wasn't expecting until like, right before surgery that I was going to wake up looking like [that].

[CW03]

The detail with which AYA cancer survivors described their physical body changes was notable as one participant described their surgical procedure as “barbaric” and that they looked like “a bear had mauled [me] with claws and then Frankenstein's monster sewed [me up] with drains coming out of [my] body with blood in them [EW02].”

For other AYAs, the cancer journey did not entail surgery or other external changes to appearance, but this did not preclude their experience of body image distress.

I think of looking at my body and how it looked before and after my cancer diagnosis and my body didn't look any different. But there were still different things going on inside of me that I wasn’t prepared for and that was a mental health battle. So, that stress and not really knowing what was going on with me internally was pretty intense and confusing.

[EW03]

#### 3.1.3 What We Gained and What We Lost

For some AYA cancer survivors, the physical manifestations of their cancer or cancer treatment evolved from a source of negative body image to a source of strength. As one participant described: “It's funny, I'm kind proud of my scars. Like it's, my scars aren't something that I'm necessarily self‐conscious about or feel ashamed about. I'm like, they're cool. Do you have this? No. [laughter] [EW04].”

Some AYA cancer survivors required a sense of regaining trust in their own body. The distrust of the body may stem from the fact that one's body was a nidus of cancer.

And I think, gosh, there are times where I feel really good and really proud of myself and proud of what my body's been able to get through. Then there are times I feel really scared and is this pain that I've been feeling for a few days? Is this metastatic cancer that is developed? Your mind goes in so many places.

[EW05]

In addition to a loss of trust in the body, some AYA cancer survivors described other unexpected losses in their lives. For one AYA, this was as simple as not fully appreciating their appearance without hair. They noted that while they “had a few wigs in rotation, once [their] eyebrows fell out, [they] looked so unfeminine, like [my] brother [EW01].”

One participant shared their realization that their role as a recognizable volunteer at their children's school was jeopardized by changes to physical appearance.

So, when she [a friend of participant’s child] saw me, like two months after my treatment ended, when I had gained a lot of weight and when I had lost my hair, she was so confused. And I'm talking to her normal, and her dad was standing right there. And then he realized like, [his child] is not realizing who I am. And it was so embarrassing.

[EW06]

#### 3.1.4 Body Image Distress as a Source of Guilt and Shame

AYA cancer survivors found it difficult at times to admit their struggles with body image distress and for some, this was in the setting of guilt as they considered other concerns that they could or “should” have. One participant shared that they felt “guilty about not liking the way [they] looked” after treatment, expressing that they should be “grateful that [they] have a body that is able to be there for [their] children [EW04].” Another participant confided that they worried that they sounded superficial in sharing thoughts on their breast reconstruction surgery.

This sounds really shallow to say, but I would show my [breast] reconstruction results to my friends, and they would say ‘that looks really good’ but for me it was disorienting and nauseating to see myself after surgery.

[CW02]

One participant shared their recollection of an early conversation with their care team and the challenges they faced in trying to reconcile “good” clinical outcomes with “bad” body image outcomes.

The first oncologist I had, they especially focused on outcomes, like, your outcomes are really good. We have really favorable numbers for you. And I was like, this treatment is killing my sex drive, making me gain weight and that disconnect was hard to wrap my head around.

[CW04]

The emotions of one participant when discussing body image distress revealed a sense of shame that this remained an active issue for them in survivorship. “I do feel like [body image distress] is unresolved and I get tearful talking about this…I should probably have addressed that, like, years ago, right? [EW07]”

#### 3.1.5 We Are a Community, but We Lack a Community

The AYA cancer survivor with body image distress often feels isolated and perhaps as if their struggles are inappropriate or abnormal when in fact, many in their cohort share similar concerns. One participant described their initial understanding of potential side effects and the emphasis placed on this:

The side effect list was so long but kind of rushed through quickly. And I was like, gosh, these side effects are going to be hitting hard and, that does not feel great.

[CW04]

In a similar way, the discussions at the conclusion of cancer therapy also avoided addressing psychosocial concerns, including body image distress.

It was sort of, I felt like I was kind of on my own to kind of deal with some of these after‐effects. And I feel like, at least from my perspective, once I finished treatment, it's like, ‘Okay, you're kind of on your own now’. And that's been hard because I'm still adjusting to the body image changes and to this new post‐cancer me in a physical sense.

[CW05]

## 4 Discussion

The thematic analysis of the rich, deep accounts of AYA cancer survivors and their evolving experience of body image distress throughout their cancer journey provides support for the themes and subthemes outlined above, and perhaps more importantly creates space for reflection and ultimately improvement upon current practices in AYA cancer care. These themes are relevant and topical as they agree with the current sentiment that persistent body image distress can impair social re‐integration post‐treatment for AYAs and that strategies to increase positive body experiences are needed [35, 36].

To address the overall approach to AYA oncological survivorship care, the authors retrospectively understood that the inclusion of a semi‐structured interview fulfilled desires expressed by participants in those very interviews, specifically those encompassed by the themes “We are a Community, but We lack a Community” and “What We Gained and What We Lost.” On reflection, the interview at the conclusion of the expressive writing program provided closure and represented an example of trauma‐informed care.

Trauma‐informed care assumes that individuals are more likely than not to have a history of trauma and acknowledges the role that trauma may play in a variety of human experiences [37, 38]. A cancer diagnosis and subsequent treatments can be considered a form of trauma, and authors A.H.Z. and V.W. did incorporate main principles of trauma‐informed care into their approach to both the interviews themselves and the prior relationship‐building that took place throughout the duration of the study. Application of trauma‐informed care along with reflexivity in research design can create safety and empowerment during data interactions and this study demonstrates this approach.

A.H.Z. and V.W.W were the only two study team members in contact with participants and made a concerted effort to use all points of contact study, including recruitment, weekly follow‐up during the writing exercises and interviews as non‐clinical, conversational and compassionate encounters in an effort to establish rapport and reliability. Of note, initial study recruitment included the administration of the Body Image Scale (BIS), a questionnaire designed to rate the clinical significance of body image distress. As such, participants were informed ahead of time of the content of the BIS and that it had the potential to induce stress and anxiety as participants recalled potentially traumatic experiences. In one instance, a participant asked to be contacted at a later time to allow for their emotional preparedness and this was met with acceptance and encouragement by the authors. This approach supports the trauma‐informed care principles of safety, voice and choice [39].

In similar support of trauma‐informed care, the interviews were structured in a way that encouraged participant storytelling and the formation of an individual narrative [40]. Open‐ended questions not only explored the participant experience of body image distress during the cancer journey but also asked participants to share their suggestions for improving the expressive writing program specifically and AYA cancer supportive care in general. Trauma‐informed care, when successful, returns a sense of control to the individual seeking that care and the interviews conducted in this study emphasized the voice and empowerment of participants to accomplish this [41].

## 5 Implications

The lessons learned in this study approach also inform suggestions for AYA survivorship care at large, most notably adopting a trauma‐informed care approach during encounters with AYA cancer survivors, whether those encounters take place in the clinical or research environment. Practical ways to achieve this care model include encouragement of narrative accounts when conducting clinical visits and obtaining patient histories all in service of prioritizing and maintaining a consistent voice while performing research activities such as recruitment calls and data collection [42, 43, 44].

The impetus for this analysis was the candor with which study participants shared their own journeys through body image distress in relationship to their cancer diagnosis and treatment. In designing this expressive writing intervention, the study team considered the possibility that recruitment would be impeded by an unwillingness to address the sensitive issues related to body image distress during a vulnerable time. However, the opposite was true, and the interviews presented herein suggest that AYA cancer survivors are seeking an outlet for further discussion. This desire is encompassed in the noted themes of “Reconciling Intellectual and Emotional Understanding of Anticipated Body Image Changes” and “We are a Community, but We Lack a Community.”

The multidisciplinary team involved in the care of the AYA with a history of cancer can utilize this knowledge to help AYAs prior to and during treatment understand the potential body image challenges that may arise in the near and distant future. One well‐known manifestation of this in survivorship occurs in the setting of fear of cancer recurrence (FCR), defined as the fear, worry, or concern about cancer returning or progressing. Prior work has established consistent positive associations between FCR and body image dissatisfaction while body checking behaviors (body part measuring, frequent mirror‐gazing) also contributed to increased FCR in AYAs in particular [45, 46]. Acknowledgment of body image distress and its manifestation in checking behaviors as well as ongoing work toward its mitigation may have broad ramifications in the survivorship experience, including reduced FCR.

When referring to a community in the cancer care space, a natural assumption is that this community involves peer support with individuals perhaps sharing a common diagnosis or treatment course. Indeed, such groups are associated with reduced anxiety and improved coping mechanisms [47, 48]. Just as AYA cancer remains underrepresented in research studies in general, so too are age and developmentally appropriate peer support groups lacking. These communities must be built and fostered, and the AYA cancer care team may represent an under‐utilized resource in building the network of AYA cancer survivors who seek support as they navigate body image distress. Participants in our study shared that worrying about body image or focusing on that source of distress felt superficial in some ways and not warranting mention to their cancer care team. If body image distress is first mentioned by that team and later supported by providing resources for mitigation, perhaps a decline in guilt and shame will follow.

## 6 Limitations

Limitations of this study include a lack of diversity within the study population, both in terms of demographics and the cancer experience. Participants were primarily white females in the upper age range of the AYA spectrum. In a reflexive context, we also acknowledge the similarity of the authors to this majority. This study is also limited by its structure in that the interview questions were designed within the context of an expressive writing intervention and were therefore limited in their scope. Future iterations of this study would benefit from broadening the included population as it is expected that more marginalized populations may have compounding oppressive factors that influence the experience of medical care in general and likely body image distress more specifically.

## 7 Conclusions

This reflexive thematic analytic approach embedded in interpretative phenomenology established the framework to deeply explore the manifestations of body image distress in AYA cancer survivors who engaged in a body image‐focused expressive writing program. The themes identified (“Reconciling Intellectual and Emotional Understanding of Anticipated Body Image Changes,” “What We Gained and What We Lost,” “Body Image Distress as a Source of Guilt and Shame,” and “We are a Community, but We Lack a Community”) provide a rich foundation for appreciating the challenges associated with both recognition and mitigation of body image distress in this unique population. Further efforts in this space will include adopting a trauma‐informed approach to AYA cancer survivorship care to better characterize these challenges and ultimately develop strategies to ensure that all psychosocial needs of AYA cancer survivors are met in an evolving healthcare landscape.

## Author Contributions

**Victoria Wytiaz:** conceptualization, data curation, formal analysis, funding acquisition, writing – original draft, writing – review and editing. **Amy Hecht‐Zizes:** conceptualization, data curation, formal analysis, writing – original draft, writing – review and editing.

## Funding

This work was supported by the Conquer Cancer Anna Braglia Endowed Young Investigator Award in Cancer Supportive Care, which was awarded to Victoria Wytiaz, MD.

## Conflicts of Interest

The authors declare no conflicts of interest.

## Data Availability

The data that support the findings of this study are available on request from the corresponding author. The data are not publicly available due to privacy or ethical restrictions.
